# New physical examination tests for lumbar spondylolisthesis and instability: low midline sill sign and interspinous gap change during lumbar flexion-extension motion

**DOI:** 10.1186/s12891-015-0551-0

**Published:** 2015-04-22

**Authors:** Kang Ahn, Hyung-Joon Jhun

**Affiliations:** Ahnkang Pain Free Hospital, CHA University, 323 Nonhyeon-Ro, 135-930 Kangnam-Ku, Seoul Republic of Korea

**Keywords:** Lumbar spine, Physical examination, Segmental instability, Spondylolisthesis, Validation

## Abstract

**Background:**

Lumbar spondylolisthesis (LS) and lumbar instability (LI) are common disorders in patients with low back or lumbar radicular pain. However, few physical examination tests for LS and LI have been reported. In the study described herein, new physical examination tests for LS and LI were devised and evaluated for their validity. The test for LS was designated “low midline sill sign”, and that for LI was designated “interspinous gap change” during lumbar flexion-extension motion.

**Methods:**

The validity of the low midline sill sign was evaluated in 96 patients with low back or lumbar radicular pain. Validity of the interspinous gap change during lumbar flexion-extension motion was evaluated in 73 patients with low back or lumbar radicular pain. The sensitivity, specificity, and positive and negative predictive values of the two tests were also investigated.

**Results:**

The sensitivity and specificity of the low midline sill sign for LS were 81.3% and 89.1%, respectively. Positive and negative predictive values of the test were 78.8% and 90.5%, respectively. The sensitivity and specificity of the interspinous gap change test for LI were 82.2% and 60.7%, respectively. Positive and negative predictive values of the test were 77.1% and 68.0%, respectively.

**Conclusions:**

The low midline sill sign and interspinous gap change tests are effective for the detection of LS and LI, and can be performed easily in an outpatient setting.

## Background

Spondylolisthesis is the anterior migration of a vertebra in relation to the vertebrae below. Low back pain, as well as pain, numbness, or weakness in the lower extremities, are symptoms clinically associated with lumbar spondylolisthesis (LS) [1]. Prevalence estimates of spondylolisthesis among females range from 6% in Taiwan to 20–25% in the United States, whereas those among males range from 3% in Taiwan and 4–8% in the United States [2]. Despite its common occurrence, few physical findings specific for the detection of LS have been reported [3]. Kalpakcioglu et al. [4] compared clinical and radiological findings from 100 patients with, and 30 patients without, LS and reported that clinical findings, such as increased lumbar lordosis and signs of slipping, were positively correlated with radiological findings. However, the validity (i.e., sensitivity and specificity) of the clinical findings of LS detection were not presented.

Lumbar instability (LI) is presumed to be a major cause of low back pain and is often an important factor in determining the surgical indications for spinal fusion with decompression [5]. It is associated with pathological mechanisms of various spinal disorders, such as spondylolisthesis [6], peridural fibrosis [7], and failed back surgery syndrome [8]. Several clinical findings have been described as symptoms and signs of LI, including patient reports of “giving away”, “locking”, and pain exacerbation with transitional activities or sustained postures [9]. Physical examination tests for LI detection have been proposed, including a prone instability test, instability catch sign, and passive lumbar extension test [10]. However, the majority of these clinical findings and tests have demonstrated a limited ability in LI diagnosis, and only a few have investigated the diagnostic accuracy of the measures [11].

In the study described herein, new physical examination tests for LS and LI were devised, and the validity of these tests was evaluated. The test for LS was designated “low midline sill sign” and that for LI was designated “interspinous gap change” during lumbar flexion-extension motion.

## Methods

### Physical examination

**Figure 1:**
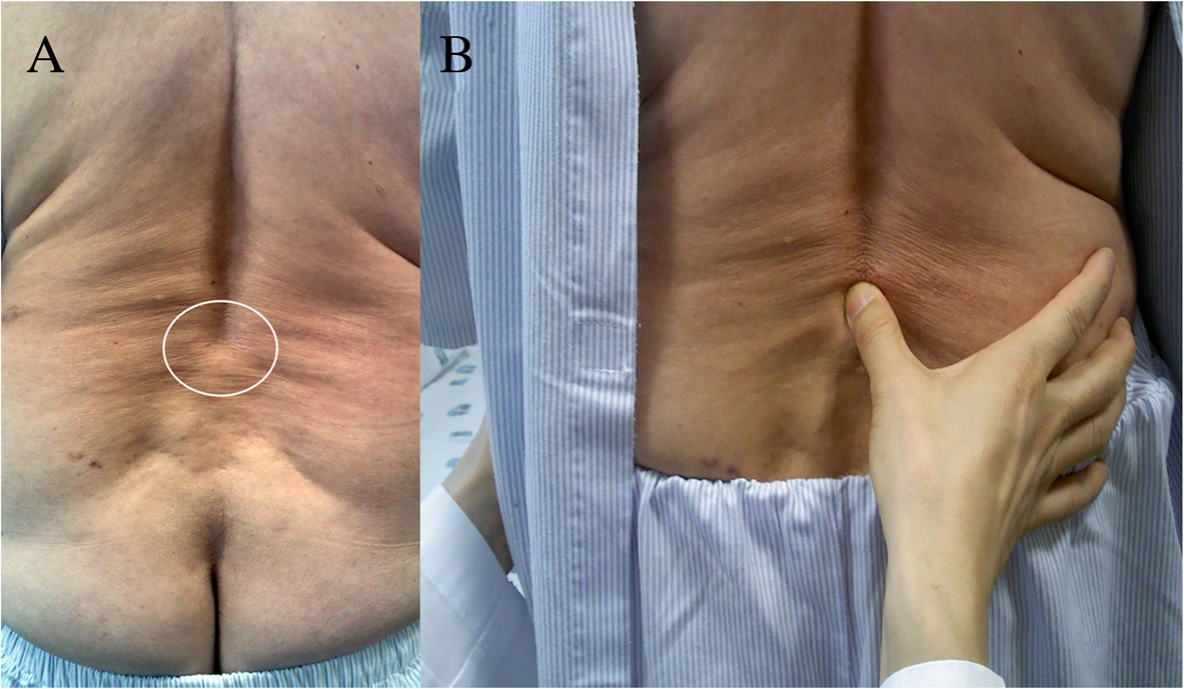
Low midline sill sign of a patient with lumbar spondylolisthesis. Inspection of the low back to detect low midline sill sign. In this case, lumbar lordosis increases and a sill like a capital “L” is observed at the L4-5 level. The skin around the sill is wrinkled and thick compared with surrounding skin **(A)**. Palpation of the low back to detect low midline sill sign. The examiner palpates the interspinous space and evaluates the position of the upper spinous process in relation to the lower spinous process **(B)**.

The LS detection test is composed of both inspection and palpation. The patient is asked to stand with his/her feet shoulder-width apart facing the examiner. The examiner inspects the midline of the patient’s back composed of spinous processes of lumbar and sacral spine cephalad-caudal direction. The sign is considered positive if lumbar lordosis increases and a sill like a capital “L” is inspected on the midline of the patient’s back. The skin around the sill is usually wrinkled and thick compared with the surrounding skin (Figure 1A). Following inspection, the midline of the patient’s back is then palpated. When an interspinous space is identified, the position of the upper spinous process in relation to the lower spinous process is evaluated. The sign is considered positive if the upper spinous process is displaced anterior to the lower spinous process and a sill like a capital “L” is palpated on the midline of the patient’s back. Tenderness is usually detected during palpation of the sill (Figure 1B).

**Figure 2:**
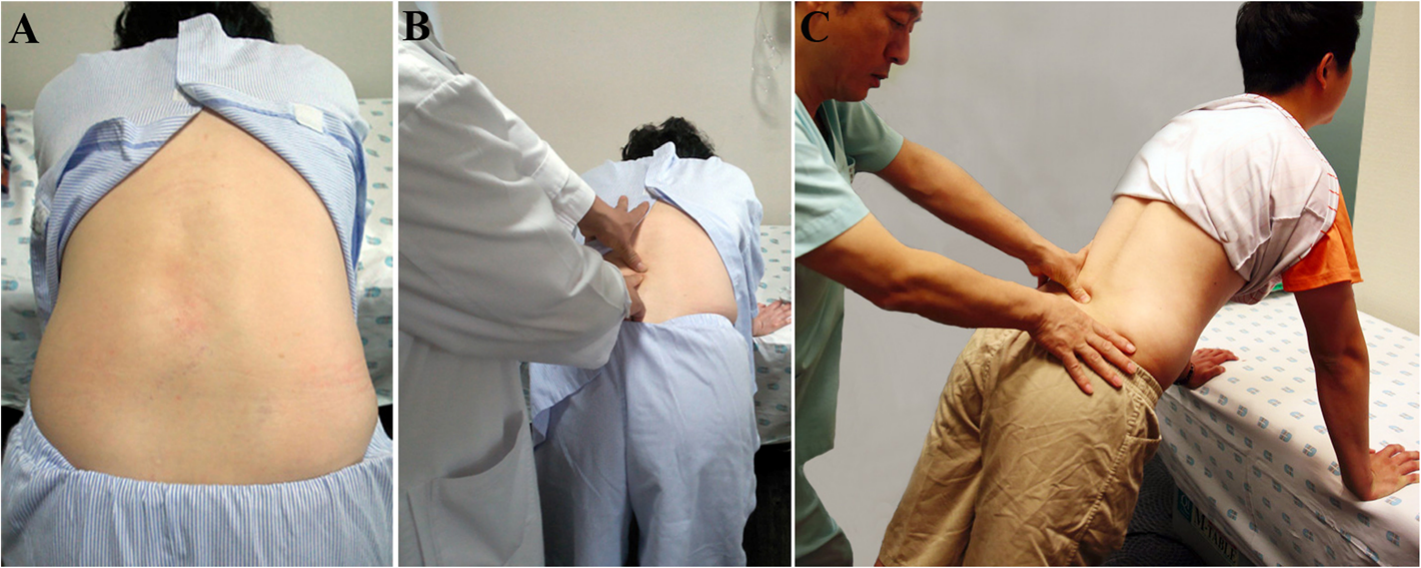
Interspinous gap change during lumbar flexion-extension motion for the detection of lumbar instability. Inspection of the low back to detect interspinous gap change. The patient is asked to stand with his/her feet shoulder-width apart, flex their back and place both hands on an examination table. The examiner inspects the patient’s back at flexion, focusing on the gaps between interspinous processes **(A)**. Palpation of the low back at flexion. The examiner palpates individual interspinous spaces of the patient’s back and evaluates the width of individual interspinous spaces and the position of the upper spinous process in relation to the lower spinous process **(B)**. Palpation of the low back at extension. The patient is asked to extend his/her upper body and push their buttocks toward the examination table as both hands are on the examination table, which reproduces lumbar extension from the flexion state. During this motion, the examiner evaluates interspinous gap change **(C)**.

The interspinous gap change during lumbar flexion-extension motion devised to detect LI is also performed in a standing position. The patient is asked to stand with his/her feet shoulder-width apart with the feet roughly leg-length from the examination table. Then the patient is asked to flex his/her back with both hands on the edge of the examination table. At flexion, the examiner inspects the patient’s back in a cranial-to-caudal direction, focusing on the gaps between the interspinous processes. If an interspinous space is bent or wider than the adjacent interspinous spaces, it is possible that this is an unstable level and particular attention should be paid to the area (Figure 2A). Following inspection, the examiner palpates the individual interspinous spaces of the patient’s back in a cranial-to-caudal direction and evaluates the width of individual interspinous spaces and the position of the upper spinous process in relation to the lower spinous process. If an interspinous space has a wider supero-inferior or antero-posterior gap between the upper and lower spinous processes than the adjacent interspinous spaces, it is suspected to be an unstable level. The interspinous space that is suspected of being unstable is selected through inspection and palpation in flexion (Figure 2B). Thereafter, the patient is asked to extend his/her upper body and push their buttocks toward the examination table with both hands on the table, which reproduces lumbar extension from a flexion state. During this motion, the examiner evaluates the change in the gap of the interspinous space that is suspected of being unstable. It is good to use both thumbs, with one placed on the interspinous space suspected of being unstable and the other placed on the interspinous space above or below that level to compare the changes in gap of the two spaces (Figure 2C). The test is considered positive if the examiner determines that the width of an interspinous space abruptly becomes narrow compared with those of other interspinous spaces, or the position of the upper spinous process in relation to the lower spinous process is changed anteriorly or posteriorly from its original state during the lumbar flexion-extension motion. Tenderness is usually detected during palpation of interspinous spaces with wide gaps, as the patient performs the flexion-extension motion.

### Subjects

Validity of the two physical examination tests was evaluated at two interventional pain management clinics in the Republic of Korea. Validity of the low midline sill sign for LS was evaluated by the corresponding author of this article (HJJ) at an interventional pain management clinic in Daejeon, Korea (Clinic A). Validity of the interspinous gap change for LI was evaluated by the first author of this article (KA) at an interventional pain management clinic in Seoul, Korea (Clinic B).

Subjects recruited to evaluate the validity of the low midline sill sign for LS detection (group A) included 96 patients with low back or lumbar radicular pain that visited Clinic A. Subjects recruited to evaluate validity of the interspinous gap change during lumbar flexion-extension motion for LI detection (group B) included 73 patients with low back or lumbar radicular pain that visited Clinic B. We excluded those who had a contraindication for a radiological evaluation, such as pregnancy; who had a history of lumbar spinal surgery; who had difficulty in standing on his/her feet; and who were unable to flex and extend the spine due to pain or muscle spasm.

The subjects were asked to rate their pain severity on a 10-point numerical rating scale (NRS), where 0 was the absence of pain and 10 was the most severe pain.

### Radiological evaluation

Presence of the low midline sill sign in group A was compared with lumbar lateral radiographs from the patients. An independent radiologist who was not informed of the physical findings evaluated the radiological findings from group A. The grade of spondylolisthesis was measured on the lateral view according to the Meyerding classification; grade I indicates a translation of the upper vertebra of up to 25% and grade II indicates that of up to 50% of the lower vertebra [12]. The type of spondylolisthesis was classified as proposed by Wiltse et al. using both lateral and oblique views [13].

The presence of an interspinous gap change during lumbar flexion-extension motion in group B was compared with lumbar flexion-extension radiographs from the patients. Another independent radiologist who was not informed of the physical findings evaluated the radiological findings of group B. Forward or backward translation of one vertebra over the other and angle of a motion segment was evaluated using flexion-extension lateral views. The cut-off between normal and abnormal movement of the spine is difficult to determine; several radiographic criteria have been proposed for LI, although there is no consensus on this issue. However, values of 10° for sagittal rotation and 4 mm for sagittal translation are typically used to infer instability [5]. Therefore, these cut-off values were used in this study.

Radiographic findings other than lumbar spondylolisthesis and instability were also reported by the radiologists at the two clinics.

### Data analysis

The sensitivity, specificity, and positive and negative predictive values of the low midline sill sign and interspinous gap change tests for LS and LI, respectively, were investigated. For data analysis, 2 × 2 tables were created from the data obtained and used to calculate sensitivity, specificity, and positive and negative predictive values.

### Ethics statement

As this study used data obtained from standard clinical and radiological examinations and no foreseeable harm was expected when obtaining data from the study subjects, written informed consent was not required from the subjects. We obtained consent for photograph from the people appearing in the photographs of this study. The study protocol was reviewed and approved by the institutional review board of Wooridul Spine Hospital (WRDIRB-Ext-2014-02).

## Results

The average age of group A was 52.8 ± 13.9 years (52.3 ± 12.6 years for men and 53.0 ± 14.6 years for women). There were 31 men and 65 women. The average self-rated pain level was 5.3 ± 1.3 points. Of the subjects, 57.3% were reported to have scoliosis and 56.3% were reported to have osteophytes in their lumbar radiographs. The average age of group B was 56.2 ± 12.4 years (59.7 ± 10.8 years for males and 55.0 ± 12.7 years for females). There were 19 men and 54 women. The average self-rated pain level was 7.1 ± 2.0 points. Of the subjects, 78.1% were reported to have disc space narrowing and 52.1% were reported to have osteophytes in their lumbar radiographs (Table 1).

**Table 1:** **Clinical data of study subjects**

	**Low midline sill sign group (Group A, n = 96)**	**Interspinous gap change group (Group B, n = 73)**
Age (years, mean ± SD)	52.8 ± 13.9	56.2 ± 12.4
Gender (M/F)	31 / 65	19 / 54
Pain level (mean ± SD)	5.3 ± 1.3	7.1 ± 2.0
Radiographic findings other than spondylolisthesis and instability (%)		
Scoliosis	55 (57.3)	14 (19.2)
Disc space narrowing	40 (41.7)	57 (78.1)
Osteophyte	54 (56.3)	38 (52.1)
Lumbarisation	6 (6.3)	2 (2.7)

Pain level was evaluated on a 10-point numeric rating scale, where 0 was no pain and 10 was the maximum severity of pain.

The low midline sill sign was validated in 32 patients (4 males and 28 females) with LS and 64 control patients (27 males and 37 females) based on their lumbar lateral radiographs. Of the 32 patients with LS, 29 (90.6%) exhibited grade I spondylolisthesis and 3 (9.4%) exhibited grade II spondylolisthesis according to the Meyerding grading system; 9 (28.1%) were classified as spondylolytic spondylolisthesis and 23 (71.9%) were classified as degenerative spondylolisthesis; 2 (6.3%) had LS at the L3-4 level, 16 (50.0%) at the L4-5 level, and 14 (43.8%) at the L5-S1 level. Of the 32 patients with LS, 26 tested positive (+) for the low midline sill sign; therefore, the sensitivity of the physical finding was 81.3%. Of the 64 patients without LS, 57 tested negative (−) for the low midline sill sign; therefore, the specificity of the physical finding was 89.1%. Positive and negative predictive values of the test were 78.8% and 90.5%, respectively (Table 2).

**Table 2:** **Diagnostic validity of the “low midline sill sign” for the detection of lumbar spondylolisthesis**

		**Radiographic lumbar spondylolisthesis**	
		**Positive**	**Negative**
Low midline sill sign	Positive	26	7
	Negative	6	57

The sensitivity, specificity, positive predictive value, and negative predictive value of the test were 81.3%, 89.1%, 78.8%, and 90.5%, respectively.

The interspinous gap change during lumbar flexion-extension motion was evaluated in 45 patients (9 males and 36 females) with LI and 28 control patients (10 males and 18 females) based on their lumbar flexion-extension radiographs. Of the 45 patients with LI, 37 tested positive (+) for interspinous gap change; therefore, the sensitivity of the test was 82.2%. Of the 28 patients without LI, 17 tested negative (−) for interspinous gap change; therefore, the specificity of the test was 60.7%. Positive and negative predictive values of the test were 77.1% and 68.0%, respectively (Table 3).

**Table 3:** **Diagnostic validity of the “interspinous gap change” during lumbar flexion-extension motion for the detection of lumbar instability**

		**Radiographic lumbar instability**	
		**Positive**	**Negative**
Interspinous gap change	Positive	37	11
	Negative	8	17

The sensitivity, specificity, positive predictive value, and negative predictive value of the test were 82.2%, 60.7%, 77.1%, and 68.0%, respectively.

## Discussion

In the study described herein, new physical examination tests for LS and LI were devised and their validity was evaluated. Validation studies revealed that the low midline sill sign showed 81.3% sensitivity and 89.1% specificity for the detection of LS. Another validation study revealed that the interspinous gap change test showed 82.2% sensitivity and 60.7% specificity for the detection of LI. We believe the low midline sill sign is highly sensitive and specific for the detection of LS and the interspinous gap change test is highly sensitive but moderately specific for the detection of LI. Therefore, these two tests are effective for LS and LI detection, and can be performed easily in an outpatient setting.

**Figure 3:**
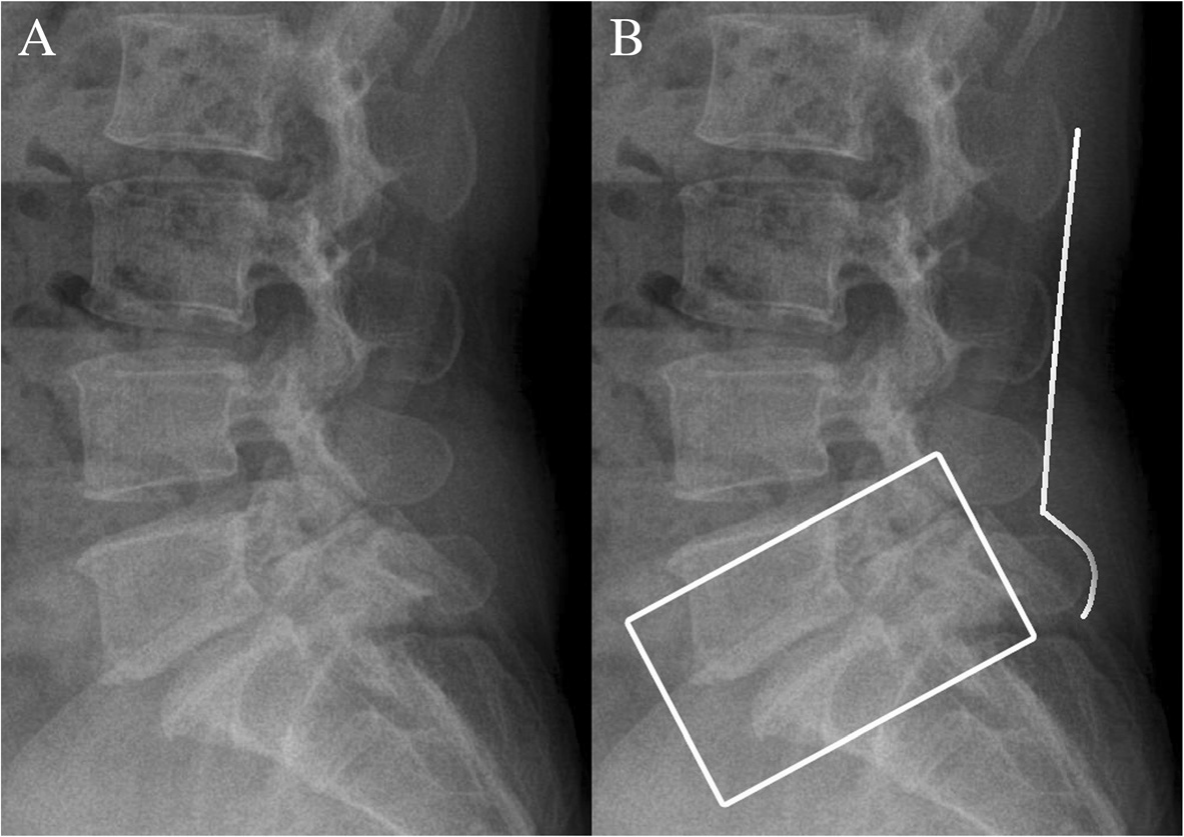
Lumbar lateral radiograph showing low midline sill. Lumbar lateral radiograph of a 47-year-old female with spondylolytic spondylolisthesis **(A)**. Explanation of the radiograph. The box indicates grade I spondylolytic spondylolisthesis at the L5-S1 level. A sill is shown between the L4-5 interspinous space when drawing a line connecting the spinous processes of the lumbar spine **(B)**.

We believe the low midline sill is formed by anterior migration of the upper spinous process in relation to the lower spinous process in LS. Figure 3A shows a lumbar lateral radiograph of a patient with L5-S1 spondylolytic spondylolisthesis. Due to spondylolysis in the pars interarticularis of the L5 vertebra, the L5 vertebral body was slipped anteriorly on the sacrum (i.e., L5-S1 spondylolisthesis) and the L5 spinous process was left behind. Therefore, a sill was formed between the L4 and L5 spinous processes (Figure 3B). These results also suggest that the sill location can be discordant with the level of LS.

**Figure 4:**
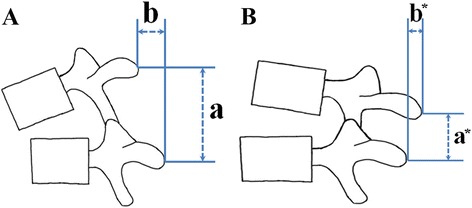
Principle of the interspinous gap change test. During flexion of the lumbar spine, the spinous process of the upper vertebra is translated superiorly and anteriorly in relation to the spinous process of the lower vertebra. “a” distance between the upper and lower spinous processes in supero-inferior direction in a flexion state; “b” distance between the upper and lower spinous processes in antero-posterior direction in a flexion state **(A)**. During extension of the lumbar spine, the spinous process of the upper vertebra is translated inferiorly and posteriorly in relation to the spinous process of the lower vertebra. “a*” distance between the upper and lower spinous processes in supero-inferior direction in an extension state; “b*” distance between the upper and lower spinous processes in antero-posterior direction in an extension state **(B)**.

Detection of a movement abnormality with passive intervertebral motion has been proposed for the detection of LI. Abbott et al. [14] performed a passive accessory intervertebral motion test and passive physiological intervertebral motion test in prone or side-lying position, and reported that the two tests are highly specific, but not sensitive. However, the interspinous gap change test is performed in an erect position, which imitates the positioning of lumbar lateral flexion-extension radiographs. Figure 4 shows the principle of the interspinous gap change test. If a patient with LI flexes his/her lumbar spine, the spinous process of the upper vertebra is translated superiorly and anteriorly in relation to the spinous process of the lower vertebra (Figure 4A). If the patient extends, the spinous process of the upper vertebra is translated inferiorly and posteriorly in relation to the spinous process of the lower vertebra (Figure 4B). Therefore, the examiner detects a position change of the upper and lower spinous processes in supero-inferior and antero-posterior direction during lumbar flexion-extension motion, which is the principle of the interspinous gap change test.

The two tests introduced in this study were devised to detect changes in the interspinous space associated with LS and LI by both inspection and palpation. Any conditions that cause difficulties in the detection of changes in the interspinous space may lead to an inaccurate physical examination. We believe disc-space narrowing and obesity are important conditions that can disturb an accurate physical examination by the two tests. Disc-space narrowing leads to a reduction in the height of the interspinous space. Obesity may also lead to disturbances by making it difficult to find interspinous spaces and any associated changes. We recommend that examiners who intend to perform these tests accumulate clinical experience through comparison of physical examination findings with radiographs and beware of conditions that may disturb an accurate physical examination.

## Conclusions

We developed two new physical examination tests for lumbar spondylolisthesis (LS) and instability (LI), and evaluated the validity of these tests. The test for LS was designated “low midline sill sign”, and that for LI was designated “interspinous gap change” during lumbar flexion-extension motion. Ninety-six patients with low back or lumbar radicular pain were recruited to test the validity of the low midline sill sign and 73 patients with low back or lumbar radicular pain were recruited to test the validity of the interspinous gap change test. The sensitivity and specificity of the low midline sill sign were 81.3% and 89.1%, respectively, whereas those of the interspinous gap change were 82.2% and 60.7%, respectively. We believe these tests are effective for the detection of both LS and LI.
